# Preparation of Spice Extracts: Evaluation of Their Phytochemical, Antioxidant, Antityrosinase, and Anti-*α*-Glucosidase Properties Exploring Their Mechanism of Enzyme Inhibition with Antibrowning and Antidiabetic Studies *In Vivo*

**DOI:** 10.1155/2022/9983124

**Published:** 2022-03-04

**Authors:** Yahya S. Alqahtani, Mater H. Mahnashi, Bandar A. Alyami, Ali O. Alqarni, Mohammed A. Huneif, Mohammed H. Nahari, Anser Ali, Qamar Javed, Hina Ilyas, Muhammad Rafiq

**Affiliations:** ^1^Department of Pharmaceutical Chemistry, College of Pharmacy, Najran University, Najran, Saudi Arabia; ^2^Pediatric Department, Medical College, Najran University, Najran, Saudi Arabia; ^3^Department of Clinical Laboratory Sciences, Najran University Najran, Saudi Arabia; ^4^Department of Zoology, Mirpur University of Science and Technology (MUST), Mirpur, 10250 AJK, Pakistan; ^5^Department of Physiology & Biochemistry, Cholistan University of Veterinary and Animal Sciences, Bahawalpur 63100, Pakistan

## Abstract

Tyrosinase and *α*-glucosidase enzymes are known as promising target candidates for inhibitors to control unwanted pigmentation and type II diabetics mellitus. Therefore, twenty extracts as enzyme inhibitors were prepared from edible spices: nutmeg, mace, star anise, fenugreek, and coriander aiming to explore their antioxidant, antibrowning, and antidiabetic potential. Results confirmed that all extracts showed potent antioxidant activity ranging from IC_50_ = 0.14 ± 0.03 to 3.69 ± 0.37 *μ*g/mL. In addition, all extracts exhibited excellent antityrosinase (IC_50_ = 1.16 ± 0.06 to 71.32 ± 4.63 *μ*g/mL) and anti-*α*-glucosidase (IC_50_4.76 ± 0.71 to 42.57 ± 2.13 *μ*g/mL) activities outperforming the corresponding standards, hydroquinone, and acarbose, respectively. Among all extracts, star anise ethyl acetate (Star anise ETAC) was found most potent inhibitor for both tyrosinase and *α*-glucosidase enzymes and was further studied to explore the mechanism of enzyme inhibition. Kinetic analysis revealed its irreversible but mixed-type tyrosinase inhibition with preferentially competitive mode of action. However, it binds reversibly with *α*-glucosidase through competitive mode of action. Further, star anise ETAC extract showed concentration dependent and posttreatment time-dependent antibrowning effect on potato slices and antidiabetic effect on diabetic rabbits *in vivo* proposing it promising candidate for tyrosinase-rooted antibrowning and *α*-glucosidase-associated diabetes management for future studies.

## 1. Introduction

Spices, a tropical herbal plant or its specific part, are not only valuable part of food but also used in fragrances, cosmetics, and medicines. Spices have been studied rigorously in recent times to understand their nature and chemical constituents. Many studies reported their important therapeutic uses, i.e., as appetizer, digestive, analgesic, carminative, hepatoprotective, blood purifier, hypolipidimic, antipyretic, antidiabetic, anti-inflammatory, antimicrobial, and antioxidant agents [1–3]. Inspired by their medicinal values, five common species of daily use, i.e., nutmeg, mace, star anise, fenugreek, and coriander, were selected. They are reported to show medicinal properties, i.e., nutmeg: astringent, stimulant, aphrodisiac, carminative, and anti-inflammatory agent; fenugreek: tonic, carminative, aphrodisiac, coriander; tonic, refrigerant, stimulant, diuretic, carminative, stomachic, analgesic, aphrodisiac, and anti-inflammatory agent [3]. These spices are commonly available and consumed in Asia; they penetrate our lives from birth to death. Therefore, we selected them to prepare the extracts for present study. This study would be the first preparing twenty extracts from five common edible spices in the region, nutmeg, mace, star anise, fenugreek, and coriander, using four solvents, methanol, ethyl acetate, n-hexane, and chloroform, comparing their antioxidant, antityrosinase, and anti-*α*-glucosidase activities identifying most potent candidate for antibrowning and diabetic potential, and having real-life applications in food and medicines.

Oxidative stress, diabetes mellitus, skin abnormal pigmentation, and fruit browning are the burning issues of this era [4–7]. Free radicals are produced during normal metabolic activities. Nearly 1/4 of inhaled oxygen is converted into free radicals. However, their excess and over accumulation have deteriorating effects on biologically important molecules like proteins, enzymes, and DNA. Although human body has well equipped and efficient antioxidant defence system, it becomes compromised when free radicals are produced higher than scavenging capacity of the body, a condition developing due to human's overdependence on synthetically processed food which ultimately requires supplements to cope [5]. Thus, excessive reactive oxygen species adversely affect the antioxidant system resulting in multiple abnormalities, i.e., neurodegenerative diseases, arthritis, cancer, diabetes mellitus, and ageing [8]. Moreover, free radical association with pigmentation and conversion of tyrosinase to dopaquinone is critical. The increased activity of free radicals in living systems leads to an increased pigmentation [9, 10], which is undesirable. Browning of fruit is another major problem occurring during storage adversely effecting its quality and posing huge economic loss [4, 6].

Tyrosinase is a key enzyme in pigment synthesis. It is responsible for skin and hair color as well as for undesirable enzymatic browning in fresh-cut fruits or plant-derived foods limiting their shelf-life with the resultant economic loss [11]. Likewise, abnormal skin pigmentation causes authentic problems, i.e., freckles, age spots, and melanoma [12]. Compounds with antioxidant and pigment inhibitory properties are desirable for skin whitening cosmetic products and for the antibrowning to maintain food quality. Spices may acquire special attention for cosmetic product improvement and to control unwanted browning of fruits as they could have less toxicity than the synthetic compounds.

In addition to pigmentation, growing scientific evidences are connecting oxidative stress with the development of diabetes and its secondary complications [13]. Oxidative stress or outrageous ROS release from autoxidation of glucose, glycated proteins, and glycation of antioxidative enzymes limit the ability to detoxify the free radicals [14]. Ultimately, high ROS load interrupts the glucose level by destroying pancreatic *β*-cells; the cells shown to have high ROS sensitivity due to their poor natural enzymatic antioxidant defence system than other body tissues, i.e., liver [15, 16]. Thus, chemicals with antioxidant properties are suggested to help in diabetic management [17]. Moreover, to control postprandial rise in blood glucose, *α*-glucosidase inhibition is critical because it delays or inhibit the carbohydrate digestion or absorption ultimately dropping the postprandial glucose level in blood [18–21]. Therefore, *α*-glucosidase inhibition is the simplest approach, and *α*-glucosidase inhibitors are attractive candidate for diabetes management [22, 23].

Thus, search for safe and potent antioxidants with tyrosinase and *α*-glucosidase inhibitory properties preferably from natural sources is desirable which may help in enzyme-associated pigmentation and diabetic management.

## 2. Materials and Method

### 2.1. Chemicals

Mushroom tyrosinase, 3,4-dihydroxyphenylalanine (L-DOPA), *α*-glucosidase from *S. cerevisiae*, acarbose, sodium carbonate, p-nitrophenyl-*α*-d-glucopyranoside (pNPG), and sodium phosphate dibasic were obtained from Sigma Aldrich and stored according to manufacturer's instructions.

### 2.2. Extract Preparation

Spices, i.e., nutmeg (*Myristica fragrans*), mace (*Myristica fragrans*), star anise (*Illicium verum*), fenugreek (*Trigonella foenum-graecum*), and coriander (*Coriandrum sativum*), were purchased from the local market, Mirpur, AJK, Pakistan. They were ground. Spices in powder form were dipped (1 : 10 g/mL) in different solvents, i.e., methanol (MeOH), ethyl acetate (ETAC), n-hexane (n-Hex), and chloroform (CHLO) for 8 days. These solvents were used because of their easy availability, easy evaporation during rotary evaporation process at low temperature, and most importantly, because of their frequent use in scientific community, availability in literature, and partitioning based on chemical solubility. Dipped samples were shacked gently two times per day. After 8 days, samples were filtered, and filtrate was evaporated by using rotary evaporator (Heidolph, Germany) at 37°C. Finally, obtained extracts were air dried at room temperature and stored at 4°C until further use.

### 2.3. Antioxidant Bioassay

Antioxidant activity using 2,2-diphenylpicrylhydrazyl (DPPH) was determined following Sharma et al. [24], with slight modifications. Briefly, 50 *μ*L DPPH and 50 *μ*L inhibitor mixture was incubated in dark for 10 min at room temperature, and then, absorbance was recorded at 490 nm wavelength. Ascorbic acid was used as standard. The experiment was performed in duplet, and IC_50_ value was calculated by using excel to compare the results.

### 2.4. Tyrosinase Inhibitory Activity

Antityrosinase assay was performed following Zaman et al. [25]. Briefly, a reaction mixture, enzyme (20 *μ*L (30 U/mL) of mushroom tyrosinase, 140 *μ*L (20 mM, pH 6.8) phosphate buffer, and 20 *μ*L of test extract, was mixed, incubated for 10 min at 37°C. Later, 20 *μ*L (0.85 mM) L-DOPA as substrate was added, incubated again for 20 min at 37°C followed by tracking dopachrome formation as measure of tyrosinase inhibition at 490 nm was checked. Each concentration was analysed in two independent experiments run in duplicate. Kojic acid was used as standard. The extent of inhibition showed by the tested extract was calculated by % inhibition formula given below, and the IC_50_ was determined by using the Microsoft Excel.

(1) $$\begin{array}{c} \text{Inhibition activity }\left(\%\right)=\frac{\left(\text{O}{\text{D}}_{\text{control}}–\text{O}{\text{D}}_{\text{sample}}\times 100\right)}{\text{O}{\text{D}}_{\text{control}}}, \end{array}$$

where OD_control_ and OD_sample_ represent the optical densities in the absence and presence of sample, respectively.

### 2.5. *α*-Glucosidase Inhibitory Activity

*α*-Glucosidase assay was performed following Umamaheswari and Sangeetha [26]. Briefly, a reaction mixture, 25 *μ*L of 0.1 M (pH 6.9) sodium phosphate buffer, 12.5 *μ*L (0.5 mM) of pNPG as substrate, 10 *μ*L extract as inhibitor, and 12.5 *μ*L of *α*-glucosidase enzyme, was mixed and incubated for 30 min at 37°C. Later, 50 *μ*L (0.2 M) sodium carbonate solution was added to terminate the reaction, and inhibition was monitored at 405 nm using microplate reader. Acarbose was used as standard, and IC_50_ value was calculated to compare with test extract results.

### 2.6. Kinetic Analysis of Tyrosinase and *α*-Glucosidase Enzyme Inhibition

To determine the mechanism of enzyme inhibition, a series of kinetic assays was performed following Zaman et al. [25] for tyrosinase and Rehman et al. [27] and Motoshima et al. [28] for *α*-glucosidase. To explore type of enzyme inhibition, Lineweaver-Burk plot (LBP) was plotted as inverse of velocities 1/*V* versus inverse of substrate concentration 1/[*S*] mM^−1^. The inhibition constant (*Ki*) was determined by two methods, the second plots of the apparent slope versus the extract concentrations, and through Dixon plot. Dixon plot was obtained by plotting different extract concentrations (as indicated in plots) versus inverse of velocities (1/*V*) with changing substrate concentrations. To check enzyme behaviour, reversible or irreversible, complexes established between extracts and enzyme were explored.

### 2.7. Phytochemical Analysis

For phytochemical analysis, qualitative tests were performed by following Chelladurai and Chinnachamy [29]. Briefly, star anise ETAC extract stock 10 mg/mL was prepared in DMSO. For flavonoid test, 125 *μ*L extract was shaken with pet ether, then dissolved in 5 mL ethanol (80%), and filtered. The filtrate was mixed with 1% KOH (1 : 1 ratio), and appearance of dark yellow colour confirmed its presence. For saponins, 250 *μ*L extract was mixed in 1 mL boiling distilled water (DI). Later, sample was cooled, mixed thoroughly, and the appearance of foam indicated its presence. For alkaloids, 125 *μ*L extract and 2 mL HCl (1%) were mixed, warmed, and filtered. The filtrate was treated with Mayer's reagent, and turbidity was noticed indicating its presence. For tannins, 125 *μ*L extract was boiled in DI (5 mL) and filtered. Few drops of FeCl_3_ (0.1%) were added, and brownish green appearance confirmed its presence. To test coumarins, 250 *μ*L extract in small tube was covered with 1 N NaOH-moistened filter paper, placed in boiling DI and examined under UV light for yellow florescence indicating its presence. To test anthocyanin and betacyanin, 1 mL extract with 2 N sodium hydroxide (500 *μ*L) was heated (3 min, 100°C), and formation of yellow colour was confirmed. For glycosides, 1 mL extract, 1.5 mL chloroform, and few drops of ammonium solution (10%) were mixed. Formation of pink colour indicated glycoside presence. For cardiac glycosides, 250 *μ*L extract, glacial acetic acid (1 mL), and few drops of ferric chloride (5%) were mixed. Later, conc. sulphuric acid (0.5 mL) was added, and the formation of brown ring at interface confirmed its presence. To test terpenoids, 0.5 mL extract, 2 mL chloroform, and conc. sulphuric acid were mixed. The formation of red brown colour confirmed its presence. For phenols, 0.5 mL extract, 1 mL DI, and few drops of ferric chloride (10%) were mixed, and the appearance of blue colour was observed. To test quinones, 0.5 mL extract and 0.5 mL conc. sulphuric acid were mixed, and the appearance of red colour confirmed its presence. To test steroids, 125 *μ*L extract, 1 mL chloroform, and 0.5 mL sulphuric acid were mixed, and the appearance of reddish brown ring confirmed steroid's presence.

### 2.8. *In Vivo* Diabetes Analysis in Rabbits

To induce diabetes and to analyse antidiabetic potential of test extract, standard method reported previously was followed [30, 31] with slight modifications. Briefly, healthy rabbits (*Oryctolagus cuniculus*) (*n* = 18) were kept in controlled area and fed on mixed vegetables, and tap water was available *ad libitum*. After habituation, diabetes was induced in 12 hr fasting rabbits (*n* = 12) by intraperitoneal administration of alloxan monohydrate (150 mg/kg of body weight) dissolved in sodium citrate buffer (4.5 pH). After 50 hrs of alloxan injection, diabetic animals (*n* = 12) were divided into 2 groups (B and C) by injecting sodium citrate (pH 4.5) in group B (*n* = 6) and by injecting test extract star anise ETAC (250 mg/kg of body weight) in group C (*n* = 6) to compare with control (untreated) group A (*n* = 6).

Group A: untreated animals

Group B: alloxan treated animals

Group C: alloxan + extract treated animals

Finally, their weight in grams (g) and blood glucose level in mg/dL were measured by blood glucose meter On Call® Plus (Acon, USA), and results were compared.

### 2.9. Antibrowning Analysis on Potato Slices

Antibrowning potential of test extract was determined following Wu et al. [32] with slight modifications. Briefly, potato slices were washed and cut into small identical slices using a slicer. They were dipped in deionized water (DI) as control and were dipped in extract star anise ETAC (1 and 1.5 mg/mL prepared in DI). Samples without any treatment were kept as negative control. All samples were placed on absorbent paper, at room temperature and photographed at 0, 2, 6, and 10 hrs to track change in colour.

### 2.10. Statistical Analysis

All experiments were performed thrice in triplicate or more. The data was organised in Microsoft Excel. To find the significance, Student's *t*-test was applied, and level of significance was expressed as *x* < 0.0001, *y* < 0.0000001, and *z* < 0.000000001.

## 3. Result and Discussion

In the present study, twenty extracts were prepared using five common spices, nutmeg, mace, star anise, fenugreek, and coriander, and four solvents, MeOH, ETAC, n-Hex, and CHLO, aiming to explore their antioxidant, antibrowning, and antidiabetic potential.

Results confirmed that all extracts possess significant antioxidant activity with IC_50_ values ranging from 0.14 ± 0.03 to 3.69 ± 0.37 *μ*g/mL (Table 1). Interestingly, 50% of total test extracts outperform the ascorbic acid, used as positive control (IC_50_ = 1.16 *μ*g/mL). Previous studies also reported the anti-oxidant activity of star anise extracts [33, 34]. Gupta et al. [35] reported antioxidant activity of nutmeg methanolic extract as IC_50_ = 1.04 mg/mL. Another study used methanolic extract of three coriander varieties, i.e., *Coriandrum sativum* L., Tunisian, Syrian, and Egyptian, and reported antioxidant activity as 27, 36, and 32 *μ*g/mL, respectively [36]. Antioxidants are shown to have close association with antibrowning [37, 38]. Browning of fruits remained a challenge in the food industry. This physiological disorder results mainly from the oxidation process leading to the formation of brown pigments which are shown to be controlled by the application of antioxidants or by reducing/inhibiting the activities of associated enzymes [37]. Thus, along antioxidant activity, we tested potential of prepared extracts to inhibit tyrosinase, key enzyme in pigmentation [39].

**Figure 1:**
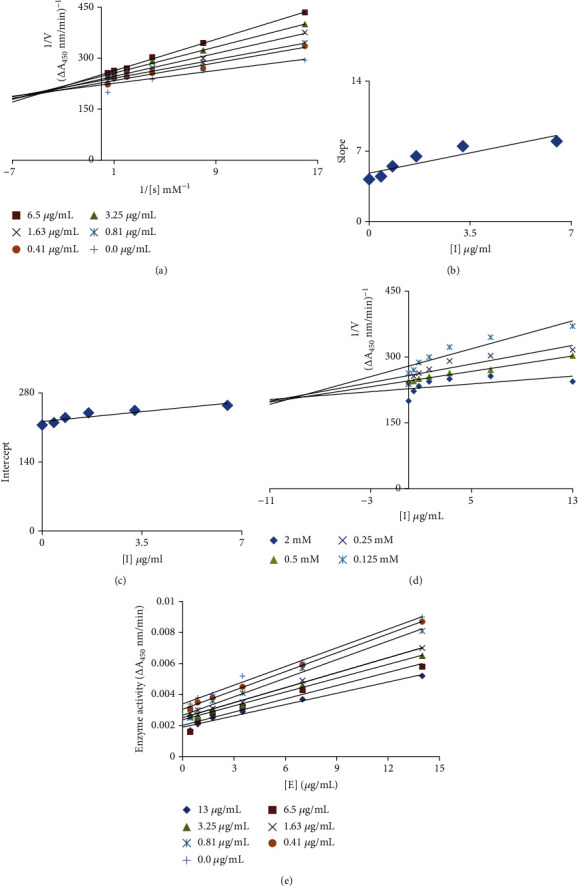
Mechanism of tyrosinase inhibition. (a) Lineweaver-Burk plot (LBP) for inhibition of tyrosinase enzyme in the presence of Star anise ETAC extract. The extract concentrations 0-13 *μ*g/mL; however, L-DOPA concentrations ranging from 0.125 to 2 mM were used. (b) The insets represent the plot of the slope from LBP versus extract. (c) The secondary replot of the LBP, 1/*V* (*y*-intercept) of versus various concentrations of extract. (d) The Dixon plot of the reciprocal of the initial velocities versus various concentrations of extract as inhibitor. (e) Relationship between the catalytic activity of L-DOPA and various concentrations of extract.

**Figure 2:**
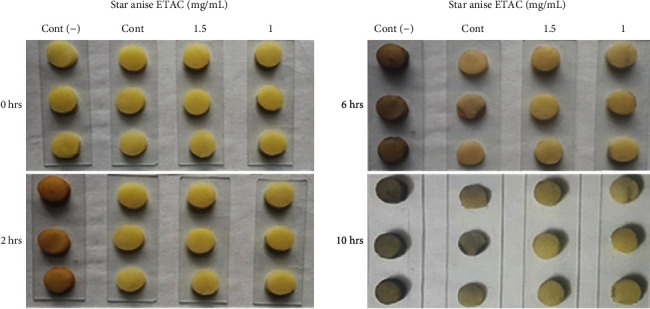
Antibrowning effect of star anise ETAC extract on potato slices. Cont (-): slices without any treatment; Cont: water; star anise ETAC extract = 1 and 1.5 mg/mL.

Analysis revealed that all extracts exhibit excellent tyrosinase inhibitory activity, IC_50_ ranging from 1.16 ± 0.06 to 71.32 ± 4.63 *μ*g/mL where all extracts outperformed the standard hydroquinone (IC_50_ = 131.34 ± 9.82 *μ*g/mL) (Table 2). Among all extracts, star anise ETAC extract was found most active tyrosinase inhibitor with lowest IC_50_ value (1.16 ± 0.06 *μ*g/mL); thus, it was selected for kinetic studies to further explore the mechanism of its enzyme inhibition and antibrowning activity. The Lineweaver-Burk plot for tyrosinase produced family of straight slopes where *V*_max_ reduces with increasing *K*_*m*_ value and by increasing concentrations of extract expressing its mixed type mode of inhibition (Figure 1(a)). In other words, star anise ETAC extract bound with free enzyme (E) as well as with enzyme-substrate (ES) complex [40]. The dissociation constant (*Ki*) and ESI dissociation constant (*Ki*′) were shown by secondary replots of slope versus extract concentration and intercept versus extract concentration, respectively (Figures 1(b) and 1(c)). The *Ki* = 8.2 *μ*g/mL and *Ki*′ = 37 *μ*g/mL for star anise ETAC indicate stronger binding with enzyme [41] that justifies its preferred competitive mode of inhibition (Table 3). Plot between catalytic activity of L-DOPA and various concentrations of Star anise ETAC extract showed irreversible mode of enzyme action (Figure 1(e)). A range of important biological activities of star anise includes antimicrobial, carminative, diuretic, and stomachic and is used in digestive disturbances, cough mixtures, and colic pain [42]. However, the present study confirms its antityrosinase activity with mechanism of enzyme inhibition. To further recapitulate tyrosinase inhibition results, further antibrowning test for star anise ETAC was performed. The visual results confirmed dose and posttreatment time-dependent antibrowning effect of star anise ETAC extract on potato slices (Figure 2). The extract-treated samples showed less browning than untreated negative control and H_2_O-treated control indicating useful application of star anise ETAC for antibrowning increasing the fruit shelf life. Thus, use of antioxidants including spices, i.e., star anise ETAC extract, can therefore be regarded as a natural antibrowning approach which helps to mitigate browning of fruits important for food and beverage industry.

**Figure 3:**
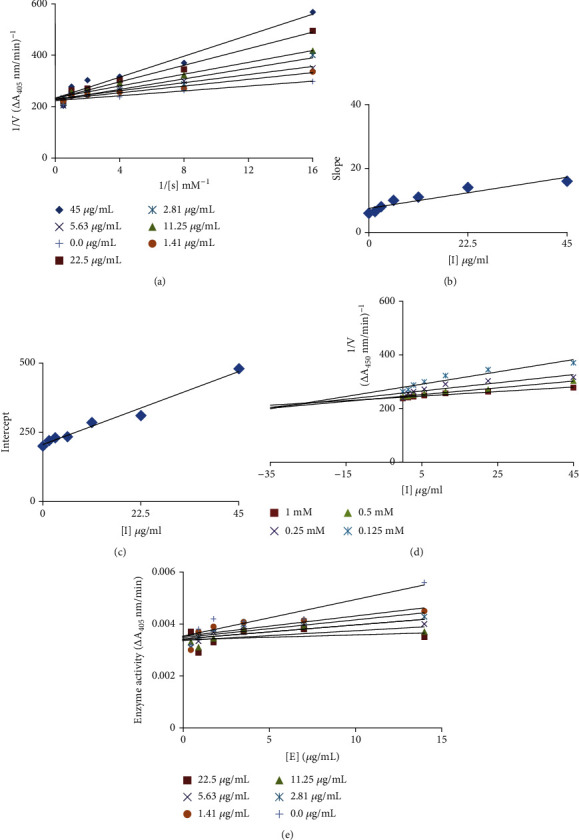
Mechanism of *α*-glucosidase inhibition. (a) Lineweaver-Burk plot (LBP) for the inhibition of *α*-glucosidase in the presence of Star anise ETAC extract. The extract concentrations 0-45 *μ*g/mL; however, pNPG concentrations (0.125 to 1 mM) were used. (b) The insets represent the plot of the slope from LBP versus inhibitor. (c) The secondary replot of the LBP, 1/*V* (*y*-intercept) of versus various concentrations of inhibitor. (d) The Dixon plot of the reciprocal of the initial velocities versus various concentrations of extract as inhibitor. (e) Relationship between the catalytic activity of pNPG and various concentrations of extract.

**Figure 4:**
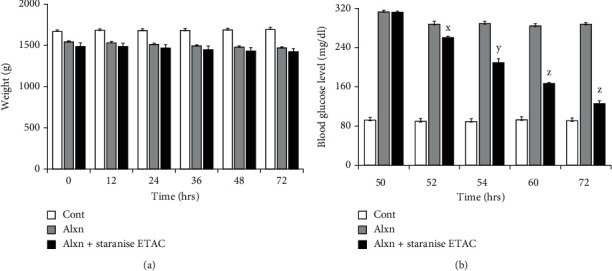
Diabetes analysis in rabbits. (a) Body weight of rabbits. (b) Measurement of blood glucose level. Experiments were performed thrice, and significance between alloxan (alxn) and alxn+Star anise ETAC was determined by Student's *t*-test as *x* < 0.0001, *y* < 0.0000001, and *z* < 0.000000001.

Likewise, *α*-glucosidase inhibitory activity of all test extracts was evaluated with potential application in diabetes management. The result confirmed the *α*-glucosidase inhibition with range of IC_50_ from 4.76 ± 0.71 *μ*g/mL to 42.57 ± 2.13 *μ*g/mL. All extracts outperformed the acarbose (IC_50_ = 201.34 ± 20.07 *μ*g/mL), used as standard (Table 2). Our results are in accordance to the report who found *Illicium verum* seed aqueous extract with *α*-glucosidase inhibitory activity as IC_50_ = 392.13 *μ*g/mL [43]. Star anise ETAC extract, like best antityrosinase activity, was found best *α*-glucosidase inhibitor with lowest IC_50_ value (4.76 ± 0.71 *μ*g/mL), thus was further explored for kinetic analysis and diabetic activity *in vivo*. The Linewever-Burrk plot produced family of straight slopes joining at *y*-axis with fixed *V*_max_ indicating competitive mode of enzyme inhibition (Figure 3(a)). The *Ki* value = 34 *μ*g/mL was obtained by secondary replot and Dixon plot (Figures 3(b) and 3(d)). Furthermore, analysis revealed reversible mode of *α*-glucosidase inhibition by star anise ETAC extract (Figure 3(e)). To recapitulate *α*-glucosidase inhibition *in vitro* results to *in vivo*, antidiabetic experiments on rabbits were performed. To induce diabetes, alloxan (alxn) was injected. The results showed no significant change in body weight among untreated, alloxan-injected, and alloxan+star anise ETAC extract-injected animals till 72 hrs (Figure 4(a)). However, star anise ETAC injection in diabetic rabbits (group C) decreases the blood glucose level significantly in time-dependent manners as compared to alloxan-induced diabetic animals (group B) (Figure 4(b)). The untreated animals (group A) showed no significant change in glucose level throughout the experiment period. The blood glucose level in group B was 313, 288, 289, 285, and 288 mg/dL, and group C was 312, 260, 209, 166, and 126 mg/dL at 50, 52, 54, 60, and 72 hrs, respectively (Figure 4(b)), indicating star anise ETAC extract as promising candidate for diabetic management.

Previous studies have shown evidences connecting oxidative stress with the development of diabetes and its secondary complications [13]. Oxidative stress or excessive ROS compromises the free radical detoxification ability of body and also interrupts the glucose level by destroying pancreatic *β*-cells [14–16]. Thus, antioxidants such as star anise ethyl acetate extract can therefore be regarded as a natural antidiabetic approach which helps improve health and blood glucose level without the use of prescription medicine.

Moreover, the phytochemical analysis of the most potent extract Star anise ETAC confirmed the presence of flavonoids, saponin, alkaloid, tannin, coumarin, anthocyanin/bethyl acetateyanin, glycoside, cardiac glycoside, terpenoid, phenol, quinone, and steroids (Table 4). Many studies have shown their tyrosinase inhibitory [44–46] and *α*-glucosidase inhibitory [47–49] effects, important for antibrowning [50] and diabetic management [22].

Thus, it is established that all extracts exhibited efficient antioxidant, antityrosinase, and anti-*α*-glucosidase activities; however, star anise ETAC extract among all was found the most potent extract. It further showed potential for tyrosinase-rooted antibrowning and *α*-glucosidase-rooted diabetic management important for food and health improvement in future.

## 4. Conclusion

The present study concluded that all twenty extracts obtained from edible spices exhibited potent antioxidant activity, important to achieve antibrowning and antidiabetic activities. In addition, all extracts exhibited excellent antityrosinase and anti-*α*-glucosidase activities outperforming their respective standards. Among all, star anise ETAC extract was found most potent inhibitor for both tyrosinase and *α*-glucosidase enzymes. Interestingly, its kinetic analysis revealed irreversible but mixed-type tyrosinase inhibition with preferentially competitive mode of action. However, it binds reversibly with *α*-glucosidase through competitive mode of action. Further, star anise ETAC extract showed concentration-dependent and posttreatment time-dependent antibrowning effect on potato slices and antidiabetic effect on diabetic rabbits *in vivo* proposing it promising candidate for tyrosinase-rooted antibrowning and *α*-glucosidase-associated diabetes management for future studies. For the future, characterization of star anise ETAC extract is suggested, and determination of key extract agent responsible for observed activities is recommended.

## Figures and Tables

**Table 1:** Antioxidant activity of prepared extracts.

DDPH assay inhibition IC_50_ ± SEM (*μ*g/mL)					
Extract	MeOH	ETAC	n-Hex		CHLO
Nutmeg	3.39 ± 0.67	1.65 ± 0.36	1.38 ± 0.27		1.19 ± 0.09
Mace	1.33 ± 0.16	0.64 ± 0.09	0.26 ± 0.1		0.59 ± 0.07
Star anise	2.67 ± 0.65	0.68 ± 0.13	3.16 ± 0.16		1.67 ± 0.17
Fenugreek	1.83 ± 0.18	0.14 ± 0.03	0.22 ± 0.02		0.41 ± 0.04
Coriander	0.7 ± 0.11	0.31 ± 0.06	3.69 ± 0.37		0.79 ± 0.12
Ascorbic acid	1.16 ± 0.06				

**Table 2:** Tyrosinase and *α*-glucosidase inhibitory activity of prepared extracts.

Extract	MeOH	ETAC	n-Hex	CHLO
	Tyrosinase inhibition IC_50_ ± SEM (*μ*g/mL)			
Nutmeg	24.66 ± 2.46	6.43 ± 0.96	1.91 ± 0.1	2.74 ± 0.1
Mace	5.91 ± 0.45	2.82 ± 0.18	2.39 ± 0.63	7.99 ± 0.6
Star anise	9.86 ± 0.84	1.16 ± 0.06	3.9 ± 0.58	2.09 ± 0.14
Fenugreek	31.23 ± 1.56	2.73 ± 0.27	19.12 ± 1	34.86 ± 1.74
Coriander	71.32 ± 4.63	10.5 ± 0.53	3.02 ± 0.3	9.65 ± 0.63
Hydroquinone	131.34 ± 9.82 (standard for tyrosinase)			
	*α*-Glucosidase inhibition IC_50_ ± SEM (*μ*g/mL)			
Nutmeg	40.65 ± 4.05	6.72 ± 0.67	23.37 ± 1.17	5.5 ± 0.55
Mace	22.31 ± 2.22	20.18 ± 2.01	16.78 ± 2.34	23.32 ± 2.21
Star anise	42.57 ± 2.13	4.76 ± 0.71	16.52 ± 1.65	9.21 ± 1.82
Fenugreek	17.76 ± 1.33	6.05 ± 0.67	15.82 ± 2.36	17.86 ± 2.22
Coriander	19.13 ± 1	25.82 ± 1.29	30.52 ± 1.92	42.57 ± 2.13
Acarbose	201.34 ± 20.07 (standard for *α*-glucosidase)			

**Table 3:** Inhibitory behaviour of most potent extract on tyrosinase and *α*-glucosidase enzymes.

Extract	Enzyme	*Ki* (*μ*g/mL)	*Ki*′ (*μ*g/mL)	Type of inhibitor	Catalytic activity
Star anise ETAC	Tyrosinase	8.2	37	Mix type	Irreversible
	*α*-Glucosidase	34	34	Competitive	Reversible

**Table 4:** Phytochemical analysis of star anise ETAC extract.

Phytochemicals tested	Star anise ETAC	Phytochemicals tested	Star anise ETAC
Flavonoids	+	Glycosides	+
Saponins	+	Cardiac glycosides	+
Alkaloids	+	Terpenoids	+
Tannins	+	Phenols	+
Coumarins	+	Quinones	+
Anthocyanin and betacyanin	+	Steroids	+

+: present.

## Data Availability

All data are included in the manuscript.
